# Clinicopathological Features and Prognosis of Papillary Thyroid Microcarcinoma for Surgery and Relationships with the BRAF^V600E^ Mutational Status and Expression of Angiogenic Factors

**DOI:** 10.1371/journal.pone.0167414

**Published:** 2016-12-09

**Authors:** Chenlei Shi, Yong Guo, Yichen Lv, Abiyasi Nanding, Tiefeng Shi, Huadong Qin, Jianjun He

**Affiliations:** 1 Department of breast surgery, the First Affiliated Hospital, Xi'an Jiao Tong University, Xi'an, China; 2 The Fourth Department of General Surgery, the Second Affiliated Hospital, Harbin Medical University, Harbin, China; 3 Department of Breast and Thyroid, Heze Municipal Hospital, Shandong Province, Heze, China; 4 The Pathology Department, the Third Affiliated Hospital, Harbin Medical University, Harbin, China; Shanghai Jiao Tong University School of Medicine, CHINA

## Abstract

**Objective:**

To investigate the clinicopathological characteristics of papillary thyroid microcarcinoma (PTMC) for surgery by comparing the difference between PTMC and larger papillary thyroid carcinoma (LPTC).

**Methods:**

We analyzed the differences in the clinicopathological characteristics, prognosis, B-type RAF kinase (BRAF)^V600E^ mutational status and expression of angiogenic factors, including pigment epithelium-derived factor (PEDF), Vascular Endothelial Growth Factor (VEGF), and hypoxia-inducible factor alpha subunit (HIF-1α), between PTMC and LPTC by retrospectively reviewing the records of 251 patients with papillary thyroid carcinoma, 169 with PTMC, and 82 with LPTC (diameter >1 cm).

**Results:**

There were no significant differences in the gender, age, multifocality, Hashimoto’s thyroiditis, TNM stage, PEDF protein expression, rate of recurrence, or mean follow-up duration between patients with PTMC or LPTC. The prevalence of extrathyroidal invasion (EI), lymph node metastasis (LNM), and BRAF mutation in patients with PTMC was significantly lower than in patients with LPTC. In addition, in PTMC patients with EI and/or LNM and/or positive BRAF (high-risk PTMC patients), the prevalence of extrathyroidal invasion, Hashimoto's disease, lymph node metastasis, tumor TNM stage, PEDF positive protein expression, the rate of recurrent disease, and the mRNA expression of anti-angiogenic factors was almost as high as in patients with larger PTC, but with no significant difference.

**Conclusions:**

Extrathyroid invasion, lymph node metastases, and BRAF^V600E^ mutation were the high risk factors of PTMC. PTMC should be considered for the same treatment strategy as LPTC when any of these factors is found. Particularly, PTMC with BRAF^V600E^ gene mutations needed earlier surgical treatment. In addition, the high cell subtype of PTMC with BRAF^V600E^ gene mutation is recommended for total thyroidectomy in primary surgery to reduce the risk of recurrence.

## Introduction

Papillary thyroid carcinoma (thyroid carcinoma papillary, PTC) is the most common pathological type of thyroid carcinoma and accounts for 80%-90% of all thyroid malignancies [1–3] with increased incidence rapidly occurring in most countries. The World Health Organization (WHO) defines papillary thyroid microcarcinoma (PTMC) as a tumor diameter of ≤ 10 mm of PTC, regardless of its invasion or lymph node metastasis and distant metastasis [4]. Previously, because PTMC could not be touched, it was difficult to find in the early stages of the disease and was occasionally found in a postoperative pathological examination of thyroid benign disease or autopsy. Thus, most PTMCs were found only because the lymph nodes or distant metastases (although it is rare) were determined. However, the asymptomatic microscopic carcinoma is still considered a progressive tumor. Therefore, the biological characteristics of PTMC tumors have gradually attracted more attention from researchers. In recent years, the frequency of newly diagnosed PTMC is increasing, probably because of the extensive development of high-frequency ultrasound and the fine needle aspiration biopsy (FNAB) technique [5]. Thyroid nodules with a diameter >3 mm can be detected by ultrasound, and the properties of the nodules can be judged by FNAB with ultrasound guidance. The viewpoint of the biological behavior of PTMC is that it tends to be benign, leading to a decrease in the attention from clinicians. Presently, there are no uniform guidelines for instructing the treatment of PTMC, making PTMC controversial regarding inadequate or excessive treatment. The latest research shows that PTMC has a high recurrence rate and can have a poor prognosis [6]. Therefore, the clinical and pathological characteristics of PTMC for surgery needs to be further determined.

The activation of oncogene B-type RAF kinase (BRAF) is the most frequent genetic event in PTC [7]. BRAF is a potent activator of the mitogen-activated protein kinase (MAPK) pathway, which plays an important role in the regulation of cell growth, proliferation, differentiation, and apoptosis [8, 9]. In recent years, several studies have shown a positive correlation between the BRAF^V600E^ mutation and several conventional high-risk clinicopathological characteristics of PTC, such as extrathyroidal invasion (EI), lymph-node metastasis (LNM), and a high disease stage [10–12], which are closely associated with cancer recurrence and a poor prognosis [13]. Pigment epithelium-derived factor (PEDF) is a 50-kDa secreted glycoprotein, first identified in cultured retinal pigment epithelial cells. It is presently one of the most promising anti-angiogenic factors, and increasing studies have shown its anti-angiogenic effects in various tumor models, such as human hepatoblastoma carcinoma, gastric carcinoma, cervical carcinoma, and papillary thyroid carcinoma [14–17]. Vascular Endothelial Growth Factor (VEGF) is a well-known pro-angiogenic factor, which has an inverse correlation with PEDF reported in various models [16–18]. The degree of oxygenation is important to neovascularization. The transcription factor hypoxia-inducible factor alpha subunit (HIF-1α) is one of the most important proteins involved in hypoxic activation. HIF-1α can activate the downstream factor VEGF, and its expression correlates with tumor progression in various carcinomas, such as pancreatic cancer, cervical carcinoma, and thyroid cancer [19–21]. These findings warrant further study of the characteristics of PTMC by analyzing the BRAF^V600E^ mutational status and expression of these anti-angiogenic factors.

In this study, we analyzed the clinicopathological characteristics, long-term prognosis, and some molecular characteristics, including BRAF^V600E^ mutation, PEDF protein expression and mRNA expression of anti-angiogenic factors. The relationships with PTMC, larger PTC (LPTC), and PTMC for surgery were further determined. These findings have implications for the development of treatment strategies for PTMC surgery to obtain a good prognosis.

## Patients and Methods

### Patients

Two hundred fifty-one patients with PTC who underwent thyroidectomy during the period from October 2014 to October 2015 at the Department of Second Affiliated Hospital of Harbin Medical University were recruited for this study. The numbers of patients diagnosed with LPTC (tumor size >1 cm) and PTMC (≤1 cm) were 169 and 82, respectively. We retrospectively reviewed the medical records, pathology reports, and subsequent clinical courses of all patients. All of the patients met the inclusion criteria, which were as follows: (1) diagnosed with papillary thyroid carcinoma (PTC) by preoperative palpation and color ultrasound equipment, and no suspicious enlarged lymph nodes were found in the cervical side; (2) confirmed as PTC by intraoperative rapid pathology and postoperative pathology detection; (3) no history of thyroid disease and not taking thyroid-related medications; (4) no history of Graves ophthalmopathy and positive for thyrotropin receptor antibody; (5) underwent thyroid resection, isthmus resection, central lymph node dissection, and postoperative follow-up.

Central neck lymph node dissection [22] refers to the dissection of all lymph adipose tissue and prelaryngeal lymph node in the front and side of the trachea and laryngeal recurrent nerve areas. The specific dissection range included all of the lymph adipose tissue in the region under the thyroid cartilage and over the sternal notch and medial area of the common carotid artery. The relevant data were collected, including demographic features (sex, age), clinical features (calcium and PTH levels, tumor size), and pathological features (multifocality, T stage, extrathyroid invasion). Clinicopathological classification was performed according to the TNM classification criteria (seventh edition, 2010) by the American Joint Committee (AJCC) on Cancer [23]. All of the operations were conducted by the same team of doctors, and histological specimens were independently reviewed by two pathologists in a blinded manner. In this study, all of the patients and their families fully understood the treatment process, written informed consent was obtained from all of the participants, and the study was approved by Ethics Committee of the Second Affiliated Hospital at Harbin Medical University.

### Cell subtype determination

PTC tissues were embedded in paraffin and were sectioned at 4 μm according to standard procedures. The sections were processed for HE staining and were used for observation by light microscopy.

### Detection of the BRAF^V600E^ mutation

Genomic DNA isolated from the primary PTC was extracted from paraffin-embedded tissues. Sections with a confirmed tumor were deparaffinized and collected for DNA extraction. The process was performed using a spinal column procedure (AmoyDx^®^ FFPE DNA Kit, Amoy Diagnostics, China) according to the manufacturer’s instructions. The absorbances of the DNA samples were measured with a spectrophotometer, and the A_260_/A_280_ values were all between 1.8 and 2.0. The DNA samples were stored at –20°C until real-time qualitative PCR analysis. The BRAF^V600E^ mutation status of each primary PTC was determined using the AmoyDx^®^ BRAF^V600E^ Mutation Detection Kit (Amoy Diagnostics, China). The mutant *BRAF* gene (encoding BRAF^V600E^) was amplified with specific primers and was detected with novel probes using Bio-Rad CFX96 (Bio-Rad Laboratories, USA) according to the manufacturer’s instructions. The carboxyfluorescein (FAM) fluorescence signal was used to evaluate the mutation status of the sample. When the sample FAM Ct value was ≥ 28, the sample was classified as negative or below the detection limit of the kit. When the sample FAM Ct value was < 28, the sample was classified as mutation positive.

### Immunohistochemical analysis

PTC tissue specimens were dissected at 4-μm intervals, deparaffinized in xylene and rehydrated in a graded series of ethanol. The slides were incubated in 3% hydrogen peroxide in distilled water for 10 min to inactivate endogenous peroxidase activity. Next, the slides underwent antigen retrieval in sodium citrate solution in a microwave oven, were blocked with 5% bovine serum albumin for 30 min, and were incubated overnight with a rabbit anti-human PEDF primary polyclonal antibody at a 1:100 dilution (Boster Bio-Engineering, China) at 4°C. The slides were then incubated with secondary antibody conjugated with biotinylated horseradish-peroxidase (Boster Bio-Engineering, China) at 37°C for 30 min. The visualization with 3, 3′-diaminobenzidine solution and counterstaining with hematoxylin were performed according to the manufacturer’s instructions. The level of PEDF was examined based on the images captured with the Olympus Imaging system (DP73, Olympus Optical Co., Ltd, Tokyo, Japan). PEDF expression was semiquantitatively categorized into three levels as follows: negative (0 points), ≤ 5% positive cells; weakly positive (1 point), 6%– 30% positive cells; moderately positive (2 points), 31%– 60% positive cells.

### Laser capture microdissection and quantitative real-time PCR (qPCR)

The frozen tissue specimens of PTMC (n = 15) and LPTC (n = 15) with EI (n = 5) and/or LNM (n = 5) and/or positive BRAF mutation (n = 5) patients in each group were used for laser capture microdissection to obtain target thyroid epithelial cells as previously described [24]; the age and sex of the participants were matched for each group. Total RNA was extracted from microdissected cells, and the cDNA converted from mRNA (DRR025A, Takara Biotechnology (Dalian) Co., Ltd. Dalian, China) was amplified by PCR to determine gene expression in microdissected cells.

The mRNA expression was analyzed using qPCR and the ABI 7500 Real-Time PCR System (Life Technology, USA) with SYBR Green I dye (Takara Biotechnology Co., Ltd. China) in accordance with the manufacturer’s protocol. The relative expression levels of *PEDF*, *VEGF* and *HIF1α* were determined using the comparative method (2^-ΔΔCt^) against the endogenous *GAPDH* controls. The information of the primers used in qPCR is shown in Table 1.

**Table 1:** Information of the primers used in Q-PCR.

Name of Gene	Sequence of primer (5’-3’)	Length of target fragment
*GAPDH*	Forward: CCACATCGCTCAGACACCAT	142 bp
	Reward: AGTTGAGGTCAATGAAGGGGT	
*PEDF*	Forward: CTCGCCATGAGATCAGCATTC	168 bp
	Reward: AGCCATAGCGTAAAACAGCCT	
*VEGF*	Forward: CTCGCCATGAGATCAGCATTC	154 bp
	Reward: AGCCATAGCGTAAAACAGCCT	
*HIF1α*	Forward: TGTCGGAGTTTGGAAAACAA	198 bp
	Reward: AAGTGGCAACTGATGAGCAA	

### Statistical analysis

All of the statistical analyses in this study were performed using SPSS 13.0 (version 13.01S; Beijing Stats Data Mining Co. Ltd, Beijing, China). Independent samples T test was used to compare the means between the groups, and χ^2^ or Fisher’s exact tests were used to compare the frequencies between the groups. The data are presented as the means ± SD or percentages, as appropriate. All P values were two-tailed, and a P value < 0.05 was considered significant for all statistical analyses in this study.

## Results

### Clinicopathological characteristics and prognosis of patients with PTC

During the period of 2014–2015, 251 patients with PTC were identified and were included in the present study. Two hundred thirty patients were female, and 21 patients were male. The mean age was 42.9 ± 10.1 years (range, 20–71). The mean size of the tumor was 1.1 ± 0.7 mm (range, 0.3–5 mm). Multifocal tumors were found in 12 patients. Twenty-five patients had extrathyroid invasion, and 84 patients had LNM. Hashimoto’s disease was observed in 40 patients. Two hundred twenty-four patients had T1 tumors, others had T3 tumors, and none had tumors in any other stage. The BRAF^V600E^ mutation was present in 69.3% of patients. In all of the samples, PEDF expression was negative in 65 patients, and positive expression was found in 74.1% of the PTC tissues—weakly positive in 121 patients and moderately positive in 65 patients. There were 6 recurrent patients. The mean follow-up duration was 45.4 ± 3.5 months (Table 2).

**Table 2:** Clinicopathologic characteristics of 251 patients with papillary thyroid carcinomas.

Characteristics	Patients (n)	Percent (%)
Gender		
Female	230	91.6
Male	21	8.4
Age, yrs	42.9 ± 10.1	
≤ 45	150	59.8
> 45	101	40.2
Tumor size, mm	1.1 ± 0.7	
≤1	169	67.3
> 1	82	32.7
Multifocality		
Single	239	95.2
Multiple (≥ 2)	12	4.8
Extrathyroid invasion		
No	226	90
Yes	25	10
Hashimoto's disease		
No	211	84.1
Yes	40	15.9
Lymph node metastases		
No	167	66.5
Yes	84	33.5
TNM stage*		
T1	224	89.2
T3	27	10.8
BRAF ^V600E^ mutation		
No	77	30.7
Yes	174	69.3
PEDF		
Negative	65	25.9
Weakly positive	121	48.2
Moderately positive	65	25.9
Recurrent		
No	245	97.6
Yes	6	2.4
Mean follow-up duration, mo	45.4 ± 3.5	

* TNM stage is based on the AJCC Cancer Staging Manual, 7th edition (2010).

### Comparison of the clinical, pathological, and prognostic features between PTMC and LPTC

There were no significant differences in the gender and age between the PTMC and LPTC groups, and the two groups showed no differences in multifocal tumors, Hashimoto's disease, tumor TNM stage, and PEDF positive expression. However, extrathyroidal invasion, lymph node metastasis, and BRAF ^V600E^ mutation were found more frequently in patients with LPTC. We also analyzed the long-term prognosis of thyroidectomy with a mean follow-up duration were approximately 45 months in all patients, and the rate of recurrent disease did not differ between the groups (Table 3).

**Table 3:** Clinicopathological characteristics in papillary thyroid carcinomas according to tumor size.

	PTMC (≤1 cm)		LPTC (>1 cm)		*P* value
	n/mean	Percent (%)	n/mean	Percent (%)	
Gender					0.58
Female	156	92.3	74	90.2	
Male	13	7.7	8	9.8	
Age, yr					0.055
≤ 45	94	55.6	56	68.3	
> 45	75	44.4	26	31.7	
Multifocality					0.347
Single	159	94.1	80	97.6	
Multiple (≥ 2)	10	5.9	2	2.4	
Extrathyroid invasion					0.03
No	157	92.9	69	84.1	
Yes	12	7.1	13	15.9	
Hashimoto's disease					0.278
No	139	82.2	72	87.8	
Yes	30	17.8	10	12.2	
Lymph node metastases					0.003
No	123	72.8	44	53.7	
Yes	46	27.2	38	46.3	
TNM stage*					0.083
1	155	91.7	69	84.1	
3	14	8.3	13	15.9	
BRAF ^V600E^ mutation					0.037
No	59	34.9	18	22	
Yes	110	65.1	64	78	
PEDF					0.08
Negative	50	29.6	15	18.3	
Weakly positive	81	47.9	40	48.8	
Moderately positive	38	22.5	27	32.9	
Recurrent					0.091
No	167	98.8	78	95.1	
Yes	2	1.2	4	4.9	
Mean follow-up duration, mo	45.6 ± 3.6		45.0 ± 3.4		0.241

* TNM stage is based on the AJCC Cancer Staging Manual, 7th edition (2010).

### Different cellular distributions in patients with PTMC or LPTC

There were significant differences in the proportion of cell subtypes between the two groups (*P* < 0.05). The proportion of typical (83.4%), columnar cell (1.2%), and high cell (1.2%) subtypes were lower in PTMC patients than in LPTC patients (typical, 90.2%; columnar cell, 2.4%; and high cell, 4.9%). The proportion of the follicular cell (10.7%) subtype was higher in PTMC patients than in LPTC patients (2.4%). The subtypes of papillary microcarcinoma and eosinophilic cells were not found in LPTC patients (Table 4).

**Table 4:** Cell subtypes in PTMC and LPTC according to tumor size.

Subtypes	PTMC		LPTC		*P* value
	n	Percent (%)	n	Percent (%)	
Typical papillary carcinoma	141	83.4	74	90.2	0.026
Follicular cell	18	10.7	2	2.4	
Papillary microcarcinoma	4	2.4	0	0	
Eosinophilic cell	2	1.2	0	0	
Columnar cell	2	1.2	2	2.4	
High cell	2	1.2	4	4.9	

### Differences in the clinicopathological characteristics and prognosis between patients with LPTC and PTMC with EI and/or LNM and/or positive BRAF ^V600E^ mutation (high-risk PTMC)

There were no significant differences between the patients with LPTC and high-risk PTMC in gender, age distribution, multifocal tumors, extrathyroidal invasion, Hashimoto's disease, lymph node metastasis, tumor TNM stages, PEDF positive expression, and rate of recurrent disease. In addition, patients with high-risk PTMC have a higher rate of BRAF^V600E^ mutation than those with LPTC (*P* < 0.05) (Table 5).

**Table 5:** Differences in the clinicopathological characteristics between high-risk PTMC and LPTC.

	High-risk PTMC		LPTC		*P* value
	n/mean	Percent (%)	n/mean	Percent (%)	
Gender					0.985
Female	110	90.2	74	90.2	
Male	12	9.8	8	9.8	
Age, yrs					0.142
≤ 45	70	57.4	56	68.3	
> 45	52	42.6	26	31.7	
Multifocality					0.321
Single	114	93.4	80	97.6	
Multiple (≥ 2)	8	6.6	2	2.4	
Extrathyroid invasion					0.199
No	110	90.2	69	84.1	
Yes	12	9.8	13	15.9	
Hashimoto's disease					0.681
No	104	85.2	72	87.8	
Yes	18	14.8	10	12.2	
Lymph node metastases					0.247
No	76	62.3	44	53.7	
Yes	46	37.7	38	46.3	
TNM stage*					0.403
1	108	88.5	69	84.1	
3	14	11.5	13	15.9	
BRAF ^V600E^ mutation					0.025
No	12	9.8	18	22	
Yes	110	90.2	64	78	
PEDF expression					0.599
Negative	27	22.1	15	18.3	
Weakly positive	62	50.8	40	48.8	
Moderately positive	33	27	27	32.9	
Recurrent					0.222
No	120	98.4	78	95.1	
Yes	2	1.6	4	4.9	
Mean follow-up duration, mo	45.9± 3.5		45.0 ± 3.4		0.067

* TNM stage is based on the AJCC Cancer Staging Manual, 7th edition (2010); High-risk PTMC: PTMC with EI and/or LNM and/or positive BRAF.

### Different cellular distributions between patients with LPTC and high-risk PTMC

There were significant differences in the proportion of cell subtypes between the two groups (*P* < 0.01). The proportion of typical (83.6%) and high cell (1.6%) subtypes was lower in high-risk PTMC patients than in LPTC patients (typical, 90.2%; and high cell, 4.9%). The proportion of follicular cells (13.1%) was higher in high-risk PTMC patients than in LPTC patients (2.4%). The subtypes of papillary microcarcinoma and eosinophilic cells were not found in LPTC patients (Table 6).

**Table 6:** Cell subtypes in high-risk PTMC and LPTC.

Subtypes	High-risk PTMC		LPTC		*P* value
	n	Percent (%)	n	Percent (%)	
Typical papillary carcinoma	102	83.6	74	90.2	0.005
Follicular cell	16	13.1	2	2.4	
Papillary microcarcinoma	0	0.0	0	0.0	
Eosinophilic cell	2	1.6	0	0.0	
Columnar cell	0	0.0	2	2.4	
High cell	2	1.6	4	4.9	

High-risk PTMC: PTMC with EI and/or LNM and/or positive BRAF.

### The similar mRNA expression of PEDF, VEGF and HIF1α in PTMC and LPTC with EI and/or LNM and/or positive BRAF mutation in each group

**Fig 1:**
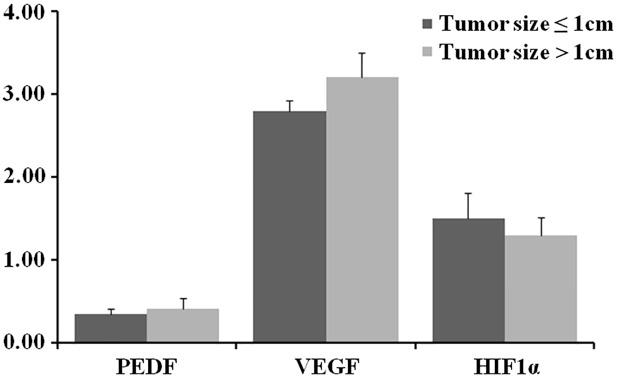
mRNA expression levels of PEDF, VEGF and HIF1α in PTMC and LPTC with EI and/or LNM and/or positive BRAF mutation in each group. EI, extrathyroidal invasion; LNM, lymph-node metastasis.

Whether the mRNA expression levels of several molecules associated with the biological behavior of PTC differed between high-risk PTMC and LPTC was determined using qPCR in frozen tissues. The results revealed that there were no significant differences in the mRNA expression levels of PEDF, VEGF and HIF1α between high-risk PTMC and LPTC samples with EI and/or LNM and/or positive BRAF mutation (Fig 1).

## Discussion

PTMC is generally considered a malignant tumor, but with a benign biological behavior, and has an excellent prognosis [25]. Brito *et al*. [26] indicated that the application of high-tech imaging technology directly leads to the increase in the PTMC diagnosis rate, but the mortality rate of thyroid cancer remains unchanged, suggesting the excessive diagnosis of thyroid cancer and even excessive treatment. However, Ito *et al*. [27] found that there was distant metastasis in the PTMC, causing the patient to have a very poor prognosis, and the recurrence rate can be as high as 20%. Now, it is confused for the deficiency or excessive treatment of PTMC. However, it is certain that the biological characteristics of PTMC are essential for the therapeutic method of treatment. Therefore, the purpose of this study was to find PTMC cases suitable for surgery and analyze the clinicopathological characteristics and prognosis.

Hay *et al*. [28] found that the recurrence rate of low-risk patients with thyroid cancer (an MACIS score less than 6 and negative lymph node metastasis) was only 3–4% after surgery for 20 years, and these patients have a good prognosis. Regarding this part of the papillary thyroid carcinoma in the dormant stage, Castro [29] et al. suggested using the term “small papillary lesions” could more accurately reflect the minimal health risks to patients. Therefore, how to make an accurate diagnosis in patients who are suitable for surgical treatment in the early stage of disease is the problem that the surgeon needs to consider and direct their efforts toward. In the present study, extrathyroidal invasion, lymph node metastasis, and BRAF ^V600E^ mutation were significantly lower in PTMC than that in larger PTC (P < 0.05). This was different from the results of Park *et al*. [30], who believed that the biological behavior of PTMC is very similar to that of PTC, and there were no significant differences in extrathyroidal invasion, lymph node metastasis, and BRAF ^V600E^ mutation between PTMC and PTC patients. Further studies on larger PTC patients and PTMC with EI and/or LNM and/or positive BRAF ^V600E^ mutation patients (high risk PTMC patients) showed that there were no significant differences between these two patient groups in gender, age distribution, multifocal tumors, extrathyroidal invasion, Hashimoto's disease, lymph node metastasis, tumor TNM stages, PEDF positive expression, and the rate of recurrent disease. Furthermore, high risk PTMC patients have a higher rate of BRAF ^V600E^ mutation than larger PTC (P < 0.05). The results suggested that the biological behavior of this high-risk PTMC is very similar to that of larger PTC; particularly, when the BRAF^V600E^ mutation is positive, the lesion requires more attention and early surgical treatment.

The BRAF^V600E^ gene mutation is currently considered a molecular marker of highly invasive PTC and is used to guide the choice of treatment and determine the prognosis [31]. The BRAF mutation rate of all patients in this study was 69.3%, and the mutation rate of PTMC (65.1%) was significantly lower than that of larger PTC (78.0%) (P < 0.05). However, the mutation rate of high-risk PTMC patients was 90.2% and was significantly higher than that of larger PTC (P < 0.05). The results suggested that BRAF^V600E^ mutation is one of the reasons that PTMC shows increased invasion; moreover, the results further indicated that the two groups differed only in tumor diameter and that there are similarities between the two groups in the mechanisms of occurrence and tumor development. A recent meta-analysis including 12 studies and involving 1168 samples showed that there is a significant relationship between the BRAF mutation and tumor cell subtypes, extrathyroidal invasion, and a high level of pathological stage. However, BRAF mutation was not associated with the size of the tumor [32], which was the same as the previous conclusions of our research group [33], further indicating that patients with PTMC, particularly high-risk PTMC, can also have a poor prognosis.

Further study on the cell subtypes of PTC showed that the constituent ratio of cell subtypes between all PTMC or high-risk PTMC and PTC were significantly different (P < 0.05), but the classical type accounts for primary weight (83.4%, 83.6% VS 90.2%) in all PTCs, which may partly explain the good prognosis of most PTC patients. In the study, we found 6 cases of high cell subtype, 2 cases of PTMC, and 4 cases of LPTC. The proportion of follicular cell in high-risk PTMC was higher than that in PTMC (13.1% VS 10.7%), and 2 cases of high cell subtypes were found in the high-risk group. During follow-up, no differences in the recurrence rate were found between the patients with high-risk PTMC and larger PTC. Two recurrent cases among PTMC patients all had high-risk PTMC, high cell subtypes, with BRAF ^V600E^ gene mutation and contralateral gland recurrence. This was similar to 4 patients in the LPTC group—all 4 had contralateral gland recurrence, and 3 patients were high cell subtypes. The results showed that the recurrence rate was significantly increased when the high cell subtype was found in the high-risk PTMC patients accompanied with gene mutations. This is consistent with Bernstein *et al* [34], who indicated that high cell PTMC is highly invasive. The present study suggested that, among high-risk group PTMC patients with BRAF^V600E^ gene mutation, high cell subtypes should undergo total thyroidectomy in the first surgery to reduce the potential risk of disease recurrence. However, for PTMC patients, the preoperative cytological examination by fine needle aspiration has the limitation of sampling quantity. Moreover, intraoperative frozen tissues can destroy the normal morphology of the tumor tissue, and these factors limit the feasibility and accuracy of early diagnosis on the subtypes of cells [35]. The American Thyroid Association Management Guidelines [36] indicated that, when patients with unilateral gland resection in initial surgery underwent contralateral side gland resection, it can be ensured that the multicentric carcinoma can be completely resected, which is beneficial to the patient for further treatment using iodine 131 therapy, and surgical risk is equivalent to the total thyroid resection in initial surgery. Therefore, for patients with BRAF^V600E^ mutation and high cell type PTMC in postoperative pathology, additional total thyroidectomy is recommended to reduce the risk of recurrence.

To further study the difference between high-risk PTMC and PTC in the biological behavior, PEDF protein expression was determined. PEDF, a multifunctional secreted glycoprotein, is one of the most promising anti-angiogenic factors at present. Many studies have shown the anti-angiogenic effects in various tumor models, such as human hepatoblastoma carcinoma, gastric carcinoma, cervical carcinoma, and papillary thyroid carcinoma [14–17]. Accumulating studies have shown that the HIF1α-VEGF pathway is activated in the progression of thyroid cancer [37,38], and PEDF could suppress tumor growth by the down-regulation of VEGF expression by inhibiting HIF1α, which showed the anti-angiogenic activity of PEDF [15–17]. However, no significant differences were found in the expression levels of PEDF by immunohistochemical analysis and PEDF, VEGF and HIF1α by mRNA analysis. The results showed that high-risk PTMC has similar characteristics in the expression of important molecular markers compared with those of LPTC, suggesting the pathogenesis of poor prognosis for the patients of PTMC with extrathyroidal invasion and/or lymph node metastasis and/or BRAF ^V600E^ mutation.

## Conclusions

In summary, the invasion of the gland, lymph node metastasis and BRAF^V600E^ gene mutation are the high-risk factors of PTMC. PTMC should be considered for the same treatment strategy as larger PTC when any factor is found. Particularly, PTMC with a BRAF^V600E^ gene mutation more often required early surgical treatment. In addition, the high cell subtype of PTMC with BRAFV^600E^ gene mutation is recommended for total thyroidectomy as the primary surgery to reduce the risk of recurrence.

## Supporting Information

S1 File Editorial Certificate of manuscript at American Journal Experts. (PDF)
